# Solid State Defect Emitters With no Electrical Activity

**DOI:** 10.1002/advs.202503350

**Published:** 2025-05-28

**Authors:** Pei Li, Song Li, Péter Udvarhelyi, Bing Huang, Adam Gali

**Affiliations:** ^1^ School of Integrated Circuit Science and Engineering Tianjin University of Technology Tianjin 300384 China; ^2^ Beijing Computational Science Research Center Beijing 100193 China; ^3^ HUN‐REN Wigner Research Centre for Physics P.O. Box 49 Budapest H‐1525 Hungary; ^4^ Department of Atomic Physics Institute of Physics Budapest University of Technology and Economics Műegyetem rakpart 3. Budapest H‐1111 Hungary; ^5^ Department of Chemistry and Biochemistry University of California Los Angeles Los Angeles CA 90095 USA; ^6^ Department of Physics Beijing Normal University Beijing 100875 China; ^7^ MTA‐WFK Lendület “Momentum” Semiconductor Nanostructures Research Group P.O. Box 49 Budapest H‐1525 Hungary

**Keywords:** first‐principles calculations, optical transition, point defects, silicon carbide, single photon emitter

## Abstract

Point defects may introduce defect levels into the fundamental bandgap of the host semiconductors that alter the electrical properties of the material. As a consequence, the in‐gap defect levels and states automatically lower the threshold energy of optical excitation associated with the optical gap of the host semiconductor. It is, therefore, a common assumption that solid‐state defect emitters in semiconductors ultimately alter the conductivity of the host. This study demonstrate, on a particular defect in 4H silicon carbide, that an unrecognized class of point defects exists that are optically active but electrically inactive in the ground state.

## Graphical abstract

Point defects may introduce in‐gap states that alter electrical properties and lower the optical excitation threshold. It is thus widely assumed that solid‐state defect emitters alter the host conductivity. This study demonstrates, on a particular defect in 4H silicon carbide, that a yet unrecognized class of point defects exists that are optically active but electrically inactive in the ground state.

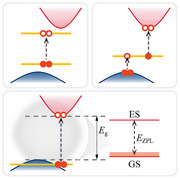

## 1 Introduction

Point defects in semiconductors play a pivotal role in determining the electrical and optical properties of the host material. Understanding the physical fundaments of point defects in semiconductors was a key to arriving at the concept of optoelectronic devices, photovoltaics and energy storage devices, and very recently, state‐of‐the‐art quantum information processing realizations,^[^ ^1^, ^2^, ^3^, ^4^, ^5^ ^]^ which have shaped the socio‐economic environment on a global scale. Point defects may introduce defect levels (DLs) within the host semiconductor's fundamental bandgap, thereby influencing its electrical conductivity, i.e., electrically active point defects.^[^ ^6^, ^7^, ^8^ ^]^ Broadly speaking, one can distinguish between so‐called shallow and deep DLs in solids, a classification that relates to the use of solids and their embedded point defects in semiconductor devices. Shallow DLs can be ionized at the typical operating temperature of semiconductor devices, whereas deep DLs remain intact. As a result, point defects introducing shallow DLs can significantly enhance the electrical conductivity of the host material. In contrast, point defects with deep DLs act as traps or recombination centers for charge carriers, temporarily or permanently reducing carrier concentration and, thus, electrical conductivity.^[^ ^6^, ^7^, ^8^ ^]^ Recombination may occur radiatively, resulting in electroluminescence. Luminescence from point defects can also be generated via appropriate illumination, which can excite both shallow and deep defects. We note that when these fluorescent point defects (i.e., color centers) are spatially isolated within the photoexcitation volume, they can act as single‐photon emitters‐key building blocks for quantum technologies. These in‐gap DLs and their associated states impact the material's optical properties by lowering the excitation threshold energy relative to the optical gap of the perfect semiconductor (see recent examples in Refs. [9, 10, 11, 12]). Therefore, a common assumption is that solid‐state defect emitters necessarily modify the host material's electrical conductivity (e.g., Refs. [13, 14, 15, 16]). We show below that this assumption is not generally valid.

**Figure 1:**
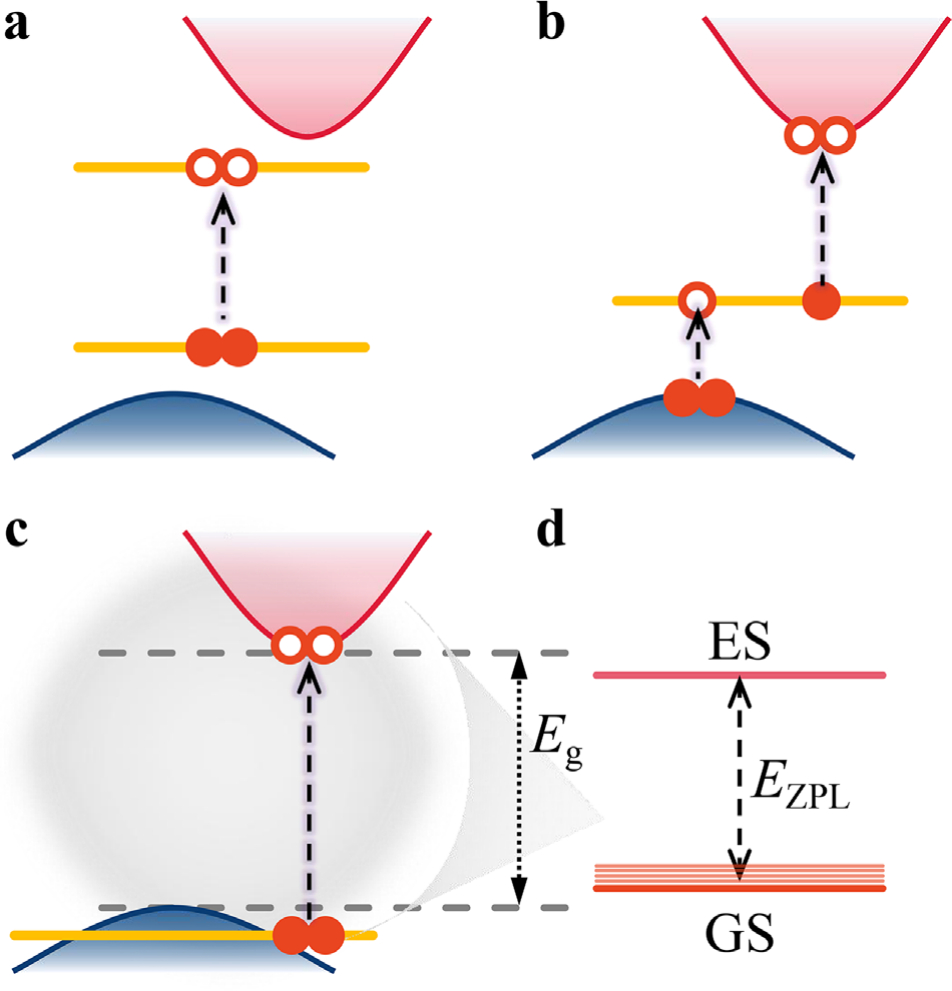
The possible optical transition mechanisms within indirect band gap semiconductors. a) Occupied and unoccupied deep DLs. b) Deep DL and band edge. c) Electrically inactive DL and band edge: establishing EIDE. d) Comparison between the values of zero‐phonon‐line (*E*_ZPL_) and band gap (*E*_g_) for the defect category in (c). *E*_ZPL_ is the energy difference between the optical excited (ES) and ground state (GS) of optical transitions with no‐phonon whereas the thinner lines are the optical transition energies when phonons are participating in the optical transition.

In **Figure** **1**, we depict the possible optical transition mechanisms within semiconductors. Many point defects introduce multiple deep DLs into the fundamental bandgap. In these defects, the optical transition could occur between the occupied and unoccupied DLs in the gap (see Figure 1a). Alternatively, the optical transition can occur between localized DLs and the band edge, either valence band maximum (VBM) or conduction band minimum (CBM) (e.g., Ref. [17]). The respective excited states may be called pseudo‐acceptor and pseudo‐donor states as they show a Rydberg‐series of the excited states converging toward the ionization threshold (see Figure 1b). We note that the optical excitation threshold energies are lower than the electrical gap between the occupied and unoccupied states participating in the optical transition because of the attracting electron‐hole interaction in the excited state. By harnessing this excitonic effect, we suggest a category of point defects that act as emitters and are electrically inactive in the ground state at the same time. A defect may introduce just one occupied DL below VBM without disturbing the bands close to VBM, so the defect is electrically inactive in the ground state and its positive charge state is not stable. This defect can be optically excited where the hole is localized in the resonant DL whereas the electron occupies a state split from CBM that builds up a pseudo‐donor excited state. The exciton binding energy in the excited state could shift the excitation energy below the optical band gap of the host semiconductor with establishing a solid state defect emitter (see Figure 1c). We label these defects as EIDE after the expression of electrically inactive defect emitters in the context.

In this study, we demonstrate the principles of EIDE on a tri‐carbon interstitial cluster in 4H silicon carbide (SiC), which produces an ultraviolet emission below the gap of 4H‐SiC. We show by first principles calculations that the given defect has zero‐phonon‐line (ZPL) emission with characteristic local vibration modes in the photoluminescence (PL) spectrum which agrees well with a previously reported defect emitter, the so‐called D_II_ center in 4H‐SiC.^[^ ^18^, ^19^, ^20^ ^]^ The optical excited state is a pseudo‐donor type and it does not show electrical activity. The effect is mediated by the attractive electron‐hole interaction in the optical excited state of the defect enhanced by the resonant defect states which is strikingly paramount in indirect semiconductors. This example unveils the EIDE category of point defects in solids. We discuss potential dopants to engineer such defects in semiconductors.

## 2 Results

### 2.1 Electronic Structure

4H‐SiC is a wide bandgap indirect semiconductor which is a platform for high‐power, high‐temperature electronic devices^[^ ^21^, ^22^, ^23^ ^]^ as well as quantum information processing^[^ ^24^, ^25^ ^]^ that makes it unique among the technologically mature semiconductors. The bandgap of 4H‐SiC is slightly reduced at elevated temperatures (see Ref. [26] and references therein). The VBM and CBM are located at the Γ‐point and the *M*‐point, respectively. The excitonic band gap of the material is 3.265 eV at 2 K,^[^ ^27^ ^]^ where the crystal phonon replica dominates the PL spectrum upon above‐bandgap illumination; nevertheless, the ZPL of the free exciton can be weakly observed too because the free exciton may gain some momentum from the defects in 4H‐SiC.^[^ ^28^ ^]^ The PL spectrum of the bound exciton of the shallow donors (nitrogen substituting carbon in the lattice at the so‐called quasicubic and hexagonal sites) can be well observed at 2 K, where the respective ZPL emissions at 3.243 eV and 3.256 eV are more pronounced as the defect potential breaks the translational symmetry of the crystal. Nevertheless, the phonon sideband still dominates in the respective PL spectrum as expected for an indirect semiconductor.^[^ ^28^ ^]^

In the following, we focus our attention to the ultraviolet (UV) D_II_ color center in 4H‐SiC recorded near cryogenic temperatures.^[^ ^18^, ^20^ ^]^ The D_II_ exhibits a sharp ZPL at 3.20 eV which is only ≈60 meV lower than that of the free exciton in 4H‐SiC.^[^ ^28^, ^29^, ^30^ ^]^ A characteristic phonon sideband was also observed in the D_II_ PL spectrum with sharp features that were associated with local vibration modes (LVMs) of the underlying defect.^[^ ^18^, ^20^ ^]^ We note that various reports on the D_II_ PL spectrum exhibit different numbers of sharp features associated with LVMs.^[^ ^18^, ^20^ ^]^ No single defect observation has yet been carried out for D_II_ center,^[^ ^31^, ^32^, ^33^, ^34^ ^]^ thus some sharp features may not belong to the defect and could overlap with the PL spectrum of other defects. Nevertheless, previous studies attempted to identify this color center by calculating the LVMs of the defect models and comparing those to the observed features in the phonon sideband.^[^ ^32^, ^33^, ^35^ ^]^ The most recent study proposed the tri‐carbon interstitial cluster as the origin of the D_II_ center which was corroborated by molecular dynamics calculations revealing the high‐temperature stability of the defect in line with the observations.^[^ ^32^ ^]^ However, the nature of optical transition has not yet been discussed for any model. Furthermore, it has been recently shown that not all the calculated LVMs show up in the observed phonon sideband of the PL spectrum of other carbon clusters in 4H‐SiC,^[^ ^17^ ^]^ thus identification of this ultraviolet color center has not yet been established.

**Figure 2:**
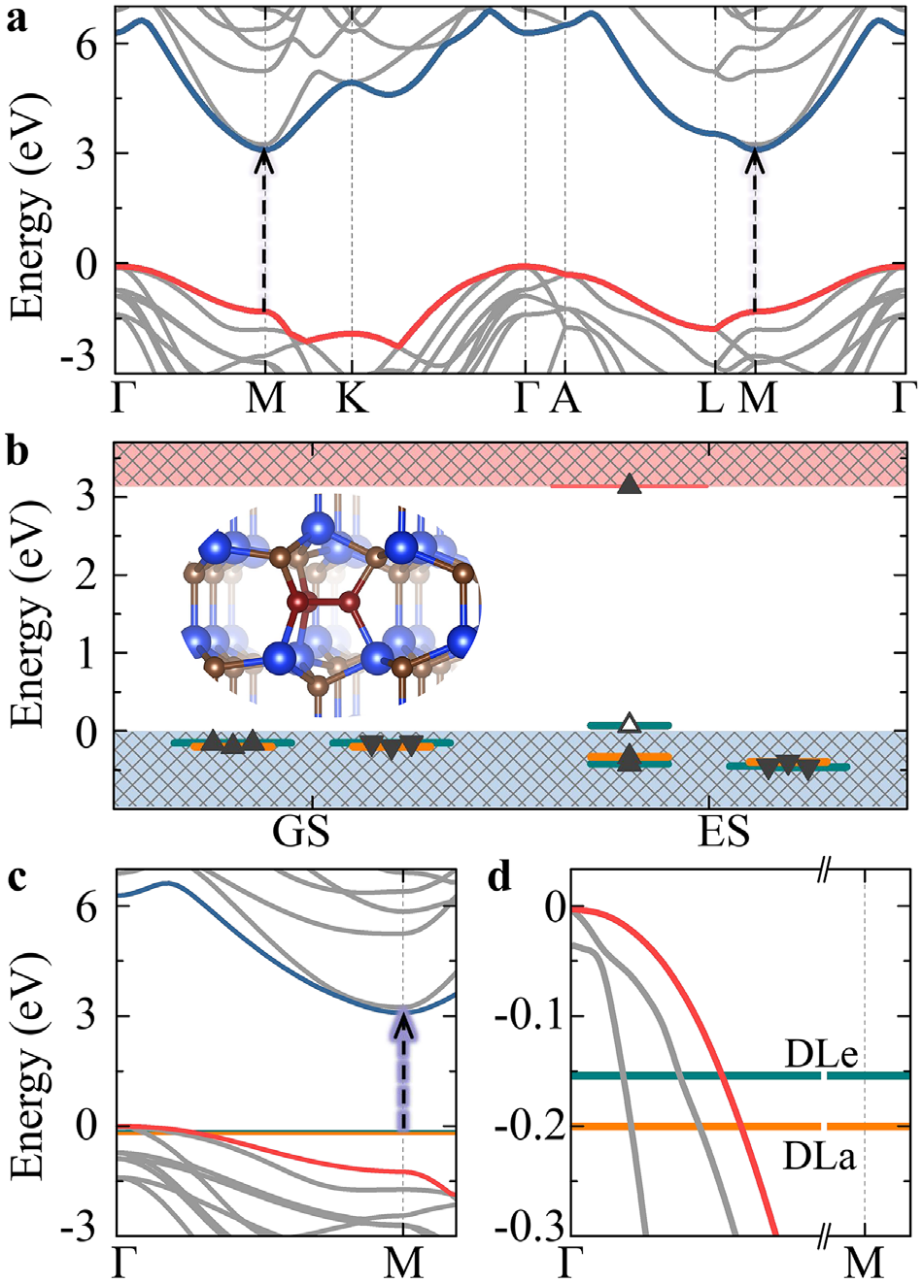
Electronic structure plots. a) The band structure of 4H‐SiC primitive cell. b) The calculated Kohn‐Sham DLs of the ground state (GS) and excited state (ES) for tri‐carbon interstitial cluster. The occupied and unoccupied DLs are labelled by filled and empty triangles, respectively. The excited states were calculated by ΔSCF method.^[^ ^36^ ^]^ Inset: the configuration of tri‐carbon interstitial cluster. c) The unfolded band structure of 4H‐SiC with tri‐carbon interstitial cluster in the supercell. d) The local enlarged drawing of (c). The degenerate *e* states (DLe) and *a*_1_ state (DLa) are labelled in green and orange, respectively.

We use the tri‐carbon interstitial cluster as a working model for the D_II_ PL center in 4H‐SiC. First, the electronic structure and possible optical transitions of the host semiconductor are analyzed. The band structure of 4H‐SiC is depicted in **Figure** **2**a. Our HSE06 calculations yield 3.17 eV electronic band gap for the host 4H‐SiC which underestimates the low‐temperature data by about 0.1 eV (see Experimental Section). The electron‐hole pair in a free exciton has a crystal momentum k_*M*_, where k_*M*_ is the momentum corresponding to the *M*‐point. Consequently, direct recombination of the electron at VBM and the hole at CBM is forbidden because of the crystal‐momentum conservation law. To conserve the crystal momentum the free exciton recombination is only possible with the assistance of another particle (or quasiparticle). Therefore, the minimum direct optical transition in 4H‐SiC occurs between the band edges at the *M*‐point, with a gap of approximately 4.41 eV, and is larger than the indirect bandgap.

The tri‐carbon interstitial cluster is composed of three carbon interstitials bridging three adjacent on‐axis Si‐C bonds at the hexagonal site (see Figure 2b), which is the most stable among all possible configurations.^[^ ^17^ ^]^ The calculated Kohn‐Sham DLs for the neutral tri‐carbon interstitial cluster are shown in Figure 2b. In the singlet ground state, a double degenerate *e* state (VBM−0.15 eV) and an *a*_1_ state (VBM−0.20 eV) appear that show no dispersion unlike the host bands (see Supplementary Figure 1). We find also resonant states in this energy region when crossed with the host bands that show up quasilocalization but heavily mixed with the host bands. No further DLs emerge in the fundamental band gap and the VBM is basically unaffected by the presence of DLs (see Figure S4, Supporting Information), implying that the defect is electrical inactive (see Figure 2c,d). Indeed, we find in our density functional theory (DFT) calculations that the positively charged defect is not stable (see Figure 5, Supporting Information).

### 2.2 Optical Spectrum

**Figure 3:**
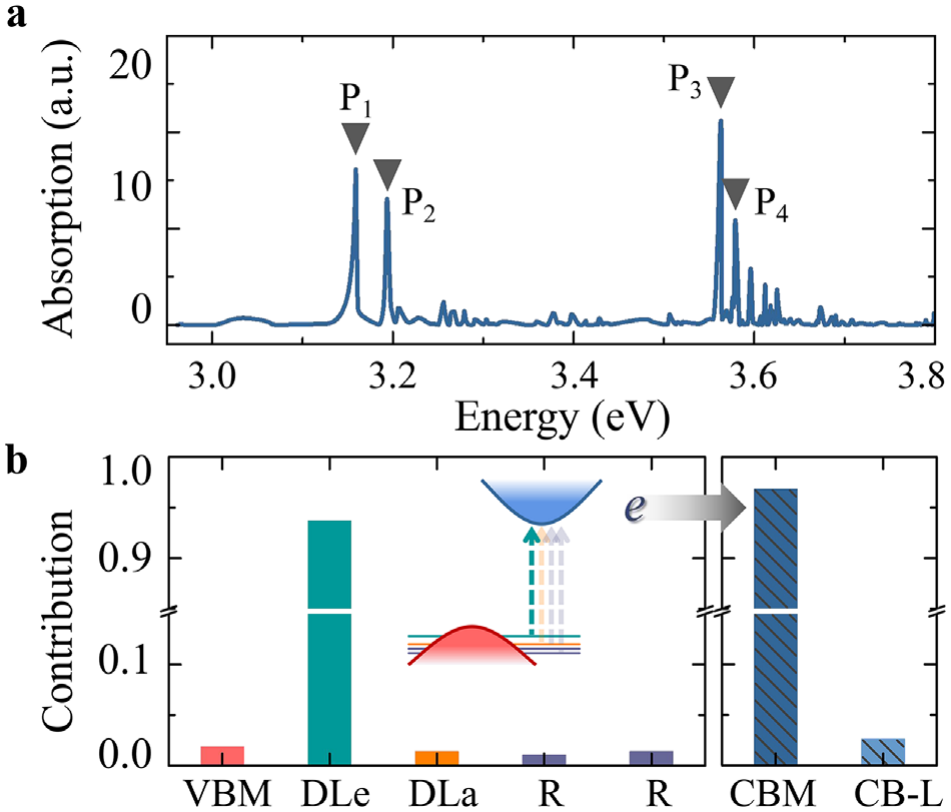
Plot and analysis of the optical spectrum. a) The optical transition intensities calculated within BSE. b) The contributions of excitation originating from VBM, DLs and resonant states (R) to CBM and conduction bands in the *L*‐point (CB‐L) for the peak P_1_ in (a). Inset: The diagram of excitation.

It can be recognized that the DLs will be well isolated from the bands around the *M*‐point, and it may be expected that the electron from the DLs can be photoexcited to the CBM that constitutes a pseudo‐donor excitonic state. We first studied the nature of the exciton and the oscillator strength of the optical transitions by a many‐body perturbation method (see Experimental Section), which allows accurate calculation of excitonic effects (see **Figure** **3**a). Here we focus to the lowest‐energy bright transitions that only play the role in the PL emission of the defect. Bright transitions occur at around 3.2 eV that we label as P_1_ and P_2_ in Figure 3a. P_1_ is double degenerate and P_2_ is non‐degenerate. In both peaks, the hole part of the exciton wavefunction is dominantly built up from the DLs and the electron part is located in the conduction bands at the *M*‐point. The contribution is about 95% and 99% for P_1_ and P_2_ peaks, respectively. In contrast to P_1_ and P_2_ peaks, the double degenerate P_3_ and P_4_ peaks in Figure 3a are mainly caused by the excitation from DLs to the conduction bands at the *L*‐point, collectively constituting approximately 87% and 82% of the total intensity (see Figure S9, Supporting Information). Here, we analyze the origin of the P_1_ peak in detail whereas the analysis of the other peaks can be found in Note S4 (Supporting Information). The calculated many‐body perturbation theory bandgap between the double degenerate *e* state (DLe) and the CBM at the *M*‐point is 3.57 eV, thus the exciton binding energy is about 0.4 eV. The contribution of the electron‐hole pairs to the P_1_ exciton is depicted in Figure 3b. The foremost contribution arises from excitation originating from the degenerate DLe to the CBM, constituting approximately 93.71% of the total intensity. The excitation from the *a* state (DLa) to CBM contributes about 1.40%. Thus, it can be concluded that the vast majority of the hole wavefunction has a localized nature whereas the electron wavefunction is split from the CBM in the low‐energy bound exciton.

Based on our findings about the lowest energy bright excited state in the BSE spectrum, we employed HSE06 ΔSCF method (see Experimental Section). This calculation makes it possible to simulate the ZPL energy and the phonon sideband in the PL spectrum. We assume that the vertical excitation energy can be well calculated by the many‐body perturbation method and then we apply finite size error corrections of the supercell method, and then the reorganization energy is subtracted as obtained by the HSE06 ΔSCF method to arrive at the final ZPL energy. We note that the ΔSCF procedure will self‐consistently change the Kohn‐Sham states and levels accordingly.

During optical excitation, an electron is promoted from the double degenerate *e* state to CBM, which gives rise to the so‐called dynamical Jahn‐Teller (JT) effect.^[^ ^37^ ^]^ Accordingly, the symmetry is reduced from *C*_3*v*_ to *C*_1*h*_. In calculating the respective optical spectrum, we use static JT distorted geometries exhibiting the global minimum energy in the adiabatic potential energy surface. In the excited state, the hole DL pops up in the band gap at 0.07 eV above VBM (see Figure 2b) in the spin‐polarized ΔSCF DFT calculation. The redistribution in the localization of the defect wavefunctions results in relatively modest forces on the ions and the defect reconstructs in the optical excited state with a reorganization energy of about 0.13 eV with reducing the symmetry of the defect. We considered the P_1_ peak in the BSE spectrum as the vertical excitation energy for which we applied finite size error corrections (adding 0.10 eV) that account for the pseudo‐donor nature of the excited state as explained in Note S5 (Supporting Information). The final simulated ZPL is at 3.13 eV, in good agreement with experimental data.

### 2.3 Phonon Sideband

**Figure 4:**
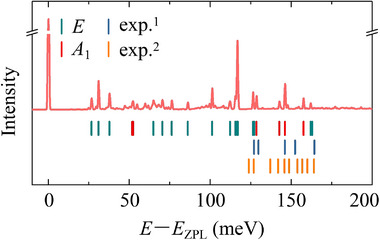
The PL spectrum of the tri‐carbon interstitial cluster. The *E* and *A*_1_ modes are marked with green and red vertical lines below the corresponding LVMs. The LVMs (blue^[^ ^18^ ^]^ and orange^[^ ^20^ ^]^ vertical lines) of D_II_ PL spectrum with frequency higher than that of 4H‐SiC bulk phonon spectrum are also listed for comparison.

We then computed the phonon sideband of the PL spectrum within Huang‐Rhys theory using the excited state's geometry as obtained by HSE06 ΔSCF method.^[^ ^36^, ^38^, ^39^ ^]^ The PL spectrum of the tri‐carbon interstitial cluster is shown in **Figure** **4** and the respective vibration frequencies are listed in Supplementary Table II. Due to symmetry reduction, both symmetry‐breaking *E* and symmetry‐conserving *A*_1_ vibrational modes contribute to the phonon sideband of the PL spectrum. The double degenerate (*E*‐mode) highest LVMs at 162 meV are stretching modes from two of the three approximately vertical C–C bonds. The third LVM is a stretching mode of the three C–C bonds, which preserves the symmetry of the defect (*A*_1_‐mode). The fourth LVM is a breathing mode of the triangle formed by the three carbon interstitials. The respective fifth and sixth LVMs result from the axial vibration of the carbon atoms right below and above the center of the tri‐carbon interstitial cluster. We conclude that the calculated ZPL energy and the LVM structure agree well with those of the D_II_ color center in 4H‐SiC (see Note S6, Supporting Information).

## 3 Discussion

We demonstrated that the D_II_ color center is a point defect which belongs to the EIDE category. In this particular case, the excited state has a pseudo‐donor nature. This explains the fact that the ZPL energy of the D_II_ center scales with the width of the band gap of SiC polytypes, c.f., Refs. [18, 20, 34]. One can generalize the feasible electronic structure of EIDE that (i) either an occupied DL should lie below, but not too far from, VBM but should be deep enough not to realize a stable positive charge state (ii) or an empty DL should lie above but not too far from CBM but high enough not to realize a stable negative charge state or (iii) the combination of both. The bound exciton state may be more easily realized in an indirect than a direct semiconductor where the attractive electron‐hole interaction of the bound exciton and the geometry relaxation in the excited state could result in a ZPL emission below the optical gap of the host semiconductor. The wavelengths of these emitters depend on the fundamental bandgap of the host semiconductors and the strength of exciton binding energies in the excited state of the defect. The last term depends on the screening effects of the material. Semiconductors with moderate and low screening can host defects with exciton binding energies that may exceed hundreds of millielectronvolts which establishes a considerable playground to realize EIDEs.

Now one may ask how EIDEs can be engineered in various materials. Electrically inactive defect in semiconductors strongly implies that (i) vacancy or vacancy complexes can be disregarded, as dangling bonds introduce levels in the fundamental band gap, (ii) the substitutional defect or dopant should be isovalent with the host atom to avoid donating or accepting electrons to the crystal, and (iii) interstitial or interstitial cluster defects should introduce even number of electrons to realize a closed‐shell singlet ground state and they should possess stronger chemical bonds than those constituting the host crystal so the occupied and unoccupied defect levels would presumably lie outside of the fundamental bandgap. Certainly, the EIDE defect models should be checked case‐by‐case in a given semiconductor. This is not surprising as this is the case, e.g., for the effective mass donor (n‐type doping) or acceptor (p‐type doping) models. Although, the usual recipe for realizing donor and acceptor states is to substitute the host atom with a dopant possessing one extra and one less electron, respectively, this recipe does not always work. As an example, the nitrogen has five valence electrons that can replace the silicon atom with four valence electrons in the silicon crystal; however, one chemical bond will be broken in the defect and the silicon dangling bond produces a deep level instead of the shallow effective mass donor. On the other hand, a boron atom with three valence electrons substituting silicon acts as an effective mass acceptor in silicon crystal despite the fact that boron and nitrogen atoms sit in the same row of the periodic table. On the contrary, nitrogen substituting the carbon in SiC indeed acts as effective mass donor as was anticipated earlier in this paper. These examples highlight the importance of accurate ab initio predictions of defect states because simple rules cannot be reliably applied for cases that are thought to be easy such as defect engineering of donors and acceptors in semiconductors. This study rather establishes the search criteria for electrically inactive defect emitters in semiconductors as quoted above. Recent advances in the theoretical spectroscopy of defects in semiconductors^[^ ^12^ ^]^ make it possible to systematically search for defects with target properties that may lead to discoveries of EIDEs, n‐type and p‐type dopants or defect qubits in semiconductors.

We further discuss the impact of EIDE defects on the electrical and optical properties of the host material. The excited state forms a bound exciton, which may act as a scattering center for charge carriers, potentially reducing conductivity and carrier mobility. This effect can be particularly relevant under high carrier concentrations, where Auger recombination becomes significant, thereby impacting the material's electrical performance.^[^ ^40^ ^]^ Such processes can occur in highly p‐type or n‐type doped conditions. While EIDE defects remain in a stable neutral charge state, an excessive concentration of free carriers could, in principle, impact their optical properties, potentially through formation of three‐particle bound exciton leading to Auger recombination and enhanced non‐radiative decay rates. Nevertheless, such effects are expected to be minimal due to the weak interaction between a neutral defect and free carriers, particularly at elevated temperatures.

For semiconductor devices with careful design of the EIDE concentration in the intrinsic region of the semiconductors, EIDEs have the potential to diminish minority carriers within the p‐i‐n junction via exciton binding and recombination through EIDEs, thereby improving the rectification characteristics of the device. Furthermore, in the realm of electroluminescence, achieving control over the recombination of bound excitons through EIDEs can lead to efficient light emission. This technique can be applied to devices such as light‐emitting diodes (LEDs). Therefore, they can be utilized in the production of UV LEDs or lasers, as well as in other applications requiring ultraviolet light, such as in biomedical and communication fields.

Additionally, due to their unique nature of being optically active yet electrically inactive, EIDEs may offer distinct advantages for quantum technologies, enabling photostable single‐photon emission when isolated. Compared to well‐known quantum emitters such as the negatively charged nitrogen‐vacancy (NV^−^) center,^[^ ^41^, ^42^, ^43^, ^44^, ^45^, ^46^, ^47^, ^48^ ^]^ group‐IV–vacancy centers in diamond,^[^ ^49^, ^50^, ^51^, ^52^, ^53^, ^54^ ^]^ divacancy and silicon‐vacancy centers in SiC,^[^ ^55^, ^56^, ^57^, ^58^, ^59^, ^60^, ^61^, ^62^, ^63^, ^64^, ^65^ ^]^ or the T‐center in silicon,^[^ ^66^, ^67^, ^68^, ^69^ ^]^ which can undergo temporary or permanent photoionization during quantum optical manipulation, EIDEs remain electrically neutral and cannot be photoionized. This inherent neutrality eliminates charge‐state instability. Moreover, since the DLs of EIDEs lie below the valence band maximum, they do not interfere with carrier transport, making them particularly suitable for integration with electronic components in quantum chip platforms.

It was noted that the illumination of single‐photon emitters can often unintentionally photoionize nearby electrically active point defects, resulting in fluctuating electric fields in their vicinity. These fluctuations lead to spectral diffusion, which can temporarily or permanently compromise the photostability and indistinguishability of quantum emitters.^[^ ^43^, ^51^, ^61^, ^62^ ^]^ In this context, a key advantage of EIDEs in quantum technologies is their inherent electrical neutrality, which ensures a more stable electromagnetic environment for nearby quantum emitters. Furthermore, their singlet ground state prevents them from generating spin noise, offering an additional benefit for preserving the coherence of nearby point defect qubits.

Defect engineering, in principle, makes it possible to isolate D_II_ centers with proper implantation and annealing strategies, as was shown for a similar tri‐interstitial defect in silicon,^[^ ^70^ ^]^ thus quantum LEDs may also be envisioned from EIDE type of defects. These findings propose an unexplored avenue for engineering semiconductor devices, suggesting the feasibility of creating defect species that offer independent control over optical and electrical functionalities within the same platform. The implications of this work extend to the design and optimization of highly integrated and miniaturized semiconductor devices that leverage both optical and electrical attributes for enhanced functionality. Because the modularity of the opto‐electronics devices can be simplified this may lead to reduced cost of production and to contribution to green technology by lowering the energy consumption of the operation of these devices.

This study has revealed a phenomenon that expands our understanding of the interplay between defect‐induced optical and electrical effects as exemplified by a tri‐carbon interstitial defect in 4H‐SiC. Unlike the conventional assumption that solid‐state defect emitters in semiconductors necessarily affect the electrical conductivity of the host, the existence of defects were demonstrated that introduce optically active but electrically inactive states. This phenomenon may be observed in rather indirect than in direct bandgap semiconductors where all the defect levels lie outside the fundamental band gap but reside very close to the band edges that may alter the minimal optical excitation energy without influencing electrical conductivity. By exploiting the attractive interaction between electrons and holes in the optical excited state, these defects can show significant optical activity without exerting any discernible impact on the electrical conductivity. This finding might pave the ways to realize new generation opto‐electronics devices.

## 4 Experimental Section

### Computational Methodology

All the first‐principles calculations were performed using density functional theory within the projector augmented wave potential plane‐wave method, as implemented in the Vienna ab initio simulation package (VASP)^[^ ^71^ ^]^ with the projector augmented wave method.^[^ ^72^ ^]^ The electron wave functions were expanded in plane‐wave basis set limited by a cutoff of 420 eV. The fully relaxed geometries were obtained by minimizing the quantum mechanical forces between the ions falling below the threshold of 0.01 eVÅ^−1^ and the self‐consistent energies converged to 10^−5^ eV.

The screened hybrid density functional of Heyd, Scuseria, and Ernzerhof (HSE06)^[^ ^73^ ^]^ was employed to calculate the electronic structure. In this approach, a fraction of nonlocal Hartree—Fock exchange was mixed with the generalized gradient approximation of Perdew, Burke, and Ernzerhof (PBE)^[^ ^74^ ^]^ with the default fraction (α = 0.25) and the inverse screening length at 0.2 Å^−1^. The calculated bandgap is 3.17 eV. The tri‐carbon interstitial cluster was embedded in a 576‐atom 4H‐SiC supercell ensuring negligible defect‐defect interaction. The single Γ‐point scheme was convergent for the k‐point sampling of the Brillouin zone (BZ). The *M*‐point and *L*‐point in the BZ were projected into the Γ‐point in this supercell due to band folding where the lowest energy conduction bands occur in these k‐points. The excited states were calculated by ΔSCF method.^[^ ^36^, ^75^, ^76^ ^]^ The details of the calculations can be found in Note S1 (Supporting Information). It was noted that the reorganization energy and the optimized geometry of the optical excited state can be calculated with ΔSCF method which is both important to predict the photoluminescence spectrum including the phonon sideband. The band structure of 4H‐SiC with the tri‐carbon interstitial cluster is calculated using the band unfolding method.^[^ ^77^, ^78^ ^]^ In the calculation, focus was on two high symmetry points, Γ and *M*, and included 30 k‐points in the line connecting the two high symmetry k‐points.

### Phonon Modes

For the phonon modes, the corresponding dynamical matrix containing the second‐order derivatives of the total energy, was calculated using the PBE^[^ ^74^ ^]^ functional where all the atoms in the supercell were enabled to vibrate. In this case, strict threshold parameters were applied for the convergence of the electronic structure (10^−6^ eV) and atomic forces (10^−3^ eVÅ^−1^) in the geometry optimization procedure. These vibration modes were applied together with the HSE06 ground state and excited state geometries to simulate the PL spectrum of the given defects within Huang‐Rhys theory.^[^ ^36^, ^39^ ^]^ This strategy worked well for deep defects in diamond (e.g., Refs. [79, 80]).

### Excitonic Effect

To accurately consider the excitonic effect, many‐body perturbation theory based on GW approximation with Bethe‐Salpeter equation (BSE)^[^ ^81^, ^82^ ^]^ were used. The supercell with 576‐atom was used in order to reach close‐to‐converged calculation for the GW method which was very computationally demanding in the VASP implementation as more than 9000 bands were included in the single‐shot G_0_W_0_ calculation. It was noted that the CBM at the *M*‐point and the conduction band at the *L*‐point are projected into the Γ‐point in the applied supercell. The energy cutoff for the response function was set to be 150 eV. The Tamm‐Dancoff approximation was used to solve BSE. The highest one hundred valence bands and one hundred lowest conduction bands were used as the basis for the excited state in the BSE procedure. The calculations were based on the HSE06 functional which resulted in the optimized geometry and the electronic structure of the neutral tri‐carbon interstitial cluster in 4H‐SiC.

## Conflict of Interest

The authors declare no conflict of interest.

## Author Contributions

P.L. carried out the calculations. P.L., S.L., P.U., B.H., and A.G. contributed to the discussion and writing the manuscript. A.G. developed the concept of electrically inactive defect emitters and led the entire scientific project.

## Supporting information

Supporting Information

## Data Availability

The data that support the findings of this study are available from the corresponding author upon reasonable request.

## References

[1] A. Polman , M. Knight , E. C. Garnett , B. Ehrler , W. C. Sinke , Science 2016, 352, aad4424. 27081076 10.1126/science.aad4424

[2] D. D. Awschalom , L. C. Bassett , A. S. Dzurak , E. L. Hu , J. R. Petta , Science 2013, 339, 1174. 23471400 10.1126/science.1231364

[3] F. A. Zwanenburg , A. S. Dzurak , A. Morello , M. Y. Simmons , L. C. Hollenberg , G. Klimeck , S. Rogge , S. N. Coppersmith , M. A. Eriksson , Rev. Mod. Phys. 2013, 85, 961.

[4] D. D. Awschalom , R. Hanson , J. Wrachtrup , B. B. Zhou , Nat. Photonics 2018, 12, 516.

[5] G. Wolfowicz , F. J. Heremans , C. P. Anderson , S. Kanai , H. Seo , A. Gali , G. Galli , D. D. Awschalom , Nat. Rev. Mater. 2021, 6, 906.

[6] S. T. Pantelides , Rev. Mod. Phys. 1978, 50, 797.

[7] H. J. Queisser , E. E. Haller , Science 1998, 281, 945. 9703502 10.1126/science.281.5379.945

[8] A. Alkauskas , M. D. McCluskey , C. G. Van de Walle , J. Appl. Phys. 2016, 119, 181101.

[9] W. Redjem , A. Durand , T. Herzig , A. Benali , S. Pezzagna , J. Meijer , A. Y. Kuznetsov , H. Nguyen , S. Cueff , J.‐M. Gérard , I. Robert‐Philip , B. Gil , D. Caliste , P. Pochet , M. Abbarchi , V. Jacques , A. Dréau , G. Cassabois , Nat. Electron. 2020, 3, 738.

[10] S. Li , G. Thiering , P. Udvarhelyi , V. Ivády , A. Gali , Nat. Commun. 2022, 13, 1210. 35260586 10.1038/s41467-022-28876-7 PMC8904548

[11] M. W. Feil , H. Reisinger , A. Kabakow , T. Aichinger , C. Schleich , A. Vasilev , D. Waldhör , M. Waltl , W. Gustin , T. Grasser , Commun. Eng. 2023, 2, 5.

[12] Á. Gali , Nanophotonics 2023, 12, 359. 39635404 10.1515/nanoph-2022-0723 PMC11501427

[13] J. Weber , W. Koehl , J. Varley , A. Janotti , B. Buckley , C. Van de Walle , D. D. Awschalom , Proc. Natl. Acad. Sci. USA 2010, 107, 8513. 20404195 10.1073/pnas.1003052107 PMC2889300

[14] L. Gordon , J. R. Weber , J. B. Varley , A. Janotti , D. D. Awschalom , C. G. Van de Walle , MRS Bull. 2013, 38, 802.

[15] Y. Ping , T. J. Smart , Nat. Comput. Sci. 2021, 1, 646. 38217204 10.1038/s43588-021-00140-w

[16] J.‐Y. Tsai , J. Pan , H. Lin , A. Bansil , Q. Yan , Nat. Commun. 2022, 13, 492. 35079005 10.1038/s41467-022-28133-x PMC8789810

[17] P. Li , P. Udvarhelyi , S. Li , B. Huang , A. Gali , Phys. Rev. B 2023, 108, 085201.

[18] S. Sridhara , D. Nizhner , R. P. Devaty , W. J. Choyke , T. Dalibor , G. Pensl , T. Kimoto , in Materials Science Forum, vol. 264, Trans Tech Publ, Switzerland 1998, pp. 493–496.

[19] J. W. Steeds , S. Furkert , W. Sullivan , G. Wagner , in Materials science forum, vol. 527, Trans Tech Publ, Switzerland 2006, pp. 473–476.

[20] W. Sullivan , J. W. Steeds , in Materials Science Forum, vol. 556, Trans Tech Publ, Switzerland 2007, pp. 319–322.

[21] S. J. Pearton , Processing of Wide Band Gap Semiconductors, Cambridge University Press, Cambridge 2013.

[22] J. Lutz , H. Schlangenotto , U. Scheuermann , R. De Doncker , *Semiconductor Power Devices*, Springer Cham, 2018.

[23] M. Shur , S. L. Rumyantsev , M. Levinshtein , SiC Materials and Devices, World Scientific, Singapore 2006.

[24] N. T. Son , C. P. Anderson , A. Bourassa , K. C. Miao , C. Babin , M. Widmann , M. Niethammer , J. Ul Hassan , N. Morioka , I. G. Ivanov , F. Kaiser , J. Wrachtrup , D. D. Awschalom , Appl. Phys. Lett. 2020, 116, 190501.

[25] A. Csóré , A. Gali , Wide Bandgap Semiconductors for Power Electronics: Materials, Devices, Applications 2021, 2, 503.

[26] E. Cannuccia , A. Gali , Phys. Rev. Mater. 2020, 4, 014601.

[27] W. J. Choyke , D. R. Hamilton , L. Patrick , Phys. Rev. 1964, 133, A1163.

[28] I. Ivanov , U. Lindefelt , A. Henry , O. Kordina , C. Hallin , M. Aroyo , T. Egilsson , E. Janzén , Phys. Rev. B 1998, 58, 13634.

[29] O. Kordina , A. Henry , J. P. Bergman , N. T. Son , W. M. Chen , C. Hallin , E. Janzén , Appl. Phys. Lett. 1995, 66, 1373.

[30] W. M. Klahold , W. J. Choyke , R. P. Devaty , in Materials Science Forum, vol. 924, Trans Tech Publ, Switzerland 2018, pp. 239–244.

[31] M. Bockstedte , A. Mattausch , O. Pankratov , Phys. Rev. B 2004, 69, 235202.

[32] C. Jiang , D. Morgan , I. Szlufarska , Phys. Rev. B 2012, 86, 144118.

[33] A. Mattausch , M. Bockstedte , O. Pankratov , Phys. B 2001, 308‐310, 656.

[34] L. Patrick , W. Choyke , J. Phys. Chem. Solids 1973, 34, 565.

[35] A. Gali , P. Deák , P. Ordejón , N. T. Son , E. Janzén , W. J. Choyke , Phys. Rev. B 2003, 68, 125201.

[36] A. Gali , E. Janzén , P. Deák , G. Kresse , E. Kaxiras , Phys. Rev. Lett. 2009, 103, 186404. 19905820 10.1103/PhysRevLett.103.186404

[37] M. Sturge , in Solid state physics, vol. 20, Elsevier, Amsterdam 1968, pp. 91–211.

[38] K. Huang , A. Rhys , Proc. R. S. London. Series A. Math. Phys. Sci. 1950, 204, 406.

[39] A. Alkauskas , B. B. Buckley , D. D. Awschalom , C. G. Van de Walle , New J. Phys. 2014, 16, 073026.

[40] A. M. Stoneham , Theory of defects in solids: electronic structure of defects in insulators and semiconductors, Oxford University Press, Oxford 2001.

[41] M. W. Doherty , N. B. Manson , P. Delaney , F. Jelezko , J. Wrachtrup , L. C. Hollenberg , Phys. Rep. 2013, 528, 1.

[42] Á. Gali , Nanophotonics 2019, 8, 1907.

[43] V. M. Acosta , C. Santori , A. Faraon , Z. Huang , K.‐M. C. Fu , A. Stacey , D. A. Simpson , K. Ganesan , S. Tomljenovic‐Hanic , A. D. Greentree , S. Prawer , R. G. Beausoleil , Phys. Rev. Lett. 2012, 108, 206401. 23003160 10.1103/PhysRevLett.108.206401

[44] P. Siyushev , H. Pinto , M. Vörös , A. Gali , F. Jelezko , J. Wrachtrup , Phys. Rev. Lett. 2013, 110, 167402. 23679637 10.1103/PhysRevLett.110.167402

[45] N. Aslam , G. Waldherr , P. Neumann , F. Jelezko , J. Wrachtrup , New J. Phys. 2013, 15, 013064.

[46] L. Razinkovas , M. Maciaszek , F. Reinhard , M. W. Doherty , A. Alkauskas , Phys. Rev. B 2021, 104, 235301.

[47] D. Wirtitsch , G. Wachter , S. Reisenbauer , M. Gulka , V. Ivády , F. Jelezko , A. Gali , M. Nesladek , M. Trupke , Phys. Rev. Res. 2023, 5, 013014.

[48] G. Thiering , A. Gali , J. Appl. Phys. 2024, 136, 084401.

[49] G. Thiering , A. Gali , Phys. Rev. X 2018, 8, 021063.

[50] S. Dhomkar , P. R. Zangara , J. Henshaw , C. A. Meriles , Phys. Rev. Lett. 2018, 120, 117401. 29601766 10.1103/PhysRevLett.120.117401

[51] B. Machielse , S. Bogdanovic , S. Meesala , S. Gauthier , M. J. Burek , G. Joe , M. Chalupnik , Y. I. Sohn , J. Holzgrafe , R. E. Evans , C. Chia , H. Atikian , M. K. Bhaskar , D. D. Sukachev , L. Shao , S. Maity , M. D. Lukin , M. Lončar , Phys. Rev. X 2019, 9, 031022.

[52] J. Görlitz , D. Herrmann , P. Fuchs , T. Iwasaki , T. Taniguchi , D. Rogalla , D. Hardeman , P.‐O. Colard , M. Markham , M. Hatano , C. Becher , npj Quantum Inf. 2022, 8, 1.

[53] G. Garcia‐Arellano , G. I. López‐Morales , N. B. Manson , J. Flick , A. A. Wood , C. A. Meriles , Adv. Sci. 2024, 11, 2308814. 10.1002/advs.202308814 PMC11165459 38475912

[54] V. Bushmakin , O. v. Berg , C. Sauerzapf , S. Jayaram , A. Denisenko , V. Vorobyov , I. Gerhardt , D. Liu , J. Wrachtrup , Two‐Photon Interference of Photons from Remote Tin‐Vacancy Centers in Diamond, 2024, http://arxiv.org/abs/2412.17539, ArXiv:2412.17539 [quant‐ph] version: 1.

[55] M. Wagner , B. Magnusson , W. M. Chen , E. Janzén , E. Sörman , C. Hallin , J. L. Lindström , Phys. Rev. B 2000, 62, 16555.

[56] N. T. Son , P. Carlsson , J. ul Hassan , E. Janzén , T. Umeda , J. Isoya , A. Gali , M. Bockstedte , N. Morishita , T. Ohshima , H. Itoh , Phys. Rev. Lett. 2006, 96, 055501. 16486945 10.1103/PhysRevLett.96.055501

[57] A. Gali , physica status solidi (b) 2011, 248, 1337.

[58] W. F. Koehl , B. B. Buckley , F. J. Heremans , G. Calusine , D. D. Awschalom , Nature 2011, 479, 84. 22051676 10.1038/nature10562

[59] D. J. Christle , P. V. Klimov , C. F. de las Casas , K. Szász , V. Ivády , V. Jokubavicius , J. Ul‐Hassan , M. Syväjärvi , W. F. Koehl , T. Ohshima , N. T. Son , E. Janzén , D. D. Awschalom , Phys. Rev. X 2017, 7, 021046.

[60] G. Wolfowicz , C. P. Anderson , A. L. Yeats , S. J. Whiteley , F. J. Heremans , D. D. Awschalom , Nat. Commun. 2017, 8, 1876. 29192288 10.1038/s41467-017-01993-4 PMC5709515

[61] P. Udvarhelyi , R. Nagy , F. Kaiser , S.‐Y. Lee , J. Wrachtrup , A. Gali , Phys. Rev. Appl. 2019, 11, 044022.

[62] R. Nagy , M. Niethammer , M. Widmann , Y.‐C. Chen , P. Udvarhelyi , C. Bonato , J. U. Hassan , R. Karhu , I. G. Ivanov , N. T. Son , J. R. Maze , T. Ohshima , n. O. Soykal , d. Gali , S.‐Y. Lee , F. Kaiser , J. Wrachtrup , Nat. Commun. 2019, 10, 1954. 31028260 10.1038/s41467-019-09873-9 PMC6486615

[63] N. T. Son , I. G. Ivanov , J. Appl. Phys. 2021, 129, 215702.

[64] A. Csóré , I. G. Ivanov , N. T. Son , A. Gali , Phys. Rev. B 2022, 105, 165108.

[65] J. Heiler , J. Körber , E. Hesselmeier , P. Kuna , R. Stöhr , P. Fuchs , M. Ghezellou , J. Ul‐Hassan , W. Knolle , C. Becher , F. Kaiser , J. Wrachtrup , npj Quantum Mater. 2024, 9, 34.

[66] G. Davies , Phys. Rep. 1989, 176, 83.

[67] L. Bergeron , C. Chartrand , A. Kurkjian , K. Morse , H. Riemann , N. Abrosimov , P. Becker , H.‐J. Pohl , M. Thewalt , S. Simmons , PRX Quantum 2020, 1, 020301.

[68] D. B. Higginbottom , F. Kimiaee Asadi , C. Chartrand , J.‐W. Ji , L. Bergeron , M. L. W. Thewalt , C. Simon , S. Simmons , PRX Quantum 2023, 4, 020308.

[69] X. Sun , W. Redjem , T. Bersans , L. Li , J. Yang , C. Babin , G. Malladi , , J. Gao , Y. Chu , X. Pan , F. Nori , , S. Kono , Nat. Commun. 2024, 15, 46643.

[70] Y. Baron , A. Durand , P. Udvarhelyi , T. Herzig , M. Khoury , S. Pezzagna , J. Meijer , I. Robert‐Philip , M. Abbarchi , J.‐M. Hartmann , V. Mazzocchi , J.‐M. Gérard , A. Gali , V. Jacques , G. Cassabois , A. Dréau , ACS photonics 2022, 9, 2337.

[71] G. Kresse , J. Furthmüller , Phys. Rev. B 1996, 54, 11169. 10.1103/physrevb.54.11169 9984901

[72] P. E. Blöchl , Phys. Rev. B 1994, 50, 17953. 10.1103/physrevb.50.17953 9976227

[73] J. Heyd , G. E. Scuseria , M. Ernzerhof , J. Chem. Phys. 2003, 118, 8207.

[74] J. P. Perdew , K. Burke , M. Ernzerhof , Phys. Rev. Lett. 1996, 77, 3865. 10062328 10.1103/PhysRevLett.77.3865

[75] R. O. Jones , O. Gunnarsson , Rev. Mod. Phys. 1989, 61, 689.

[76] J. Gavnholt , T. Olsen , M. Engelund , J. Schiøtz , Phys. Rev. B Condens. Matter Mater. Phys. 2008, 78, 075441.

[77] V. Popescu , A. Zunger , Phys. Rev. Lett. 2010, 104, 236403. 20867256 10.1103/PhysRevLett.104.236403

[78] V. Popescu , A. Zunger , Phys. Rev. B 2012, 85, 085201.

[79] G. Thiering , A. Gali , Phys. Rev. B 2017, 96, 081115.

[80] G. Thiering , A. Gali , Phys. Rev. X 2018, 8, 021063.

[81] M. Shishkin , G. Kresse , Phys. Rev. B 2006, 74, 035101.

[82] M. Shishkin , M. Marsman , G. Kresse , Phys. Rev. Lett. 2007, 99, 246403. 18233465 10.1103/PhysRevLett.99.246403

