# Supplementary material for: Solid State Defect Emitters With no Electrical Activity
Source: Adv Sci (Weinh). 2025 May 28;12(30):e03350. doi: 10.1002/advs.202503350 (PMC12376652; doi:10.1002/advs.202503350)
Supplement: Supplementary file 1 — Supporting Information [file ADVS-12-e03350-s001.pdf]

ADVANCED SCIENCE
Open Access

Supporting Information

for *Adv. Sci.*, DOI 10.1002/advs.202503350

Solid State Defect Emitters With no Electrical Activity

*Pei Li, Song Li, Péter Udvarhelyi, Bing Huang and Adam Gali**

Supplementary Information

Solid state defect emitters with no electrical activity

Pei Li,[1,2,3] Song Li,[3,2] Péter Udvarhelyi,[3,4,5] Bing Huang,[2,6] and Adam Gali[3,4,7]

[1]*School of Integrated Circuit Science and Engineering, Tianjin University of Technology, Tianjin 300384, China*

[2]*Beijing Computational Science Research Center, Beijing 100193, China*

[3]*HUN-REN Wigner Research Centre for Physics, P.O. Box 49, H-1525 Budapest, Hungary*

[4]*Department of Atomic Physics, Institute of Physics, Budapest University of Technology and Economics, Műegyetem rakpart 3., H-1111 Budapest, Hungary*

[5]*Department of Chemistry and Biochemistry, University of California Los Angeles, Los Angeles, California 90095, United States*

[6]*Department of Physics, Beijing Normal University, Beijing, 100875, China*

[7]*MTA-WFK Lendület "Momentum" Semiconductor Nanostructures Research Group, P.O. Box 49, H-1525 Budapest, Hungary*

SUPPLEMENTARY NOTE 1: THE WAVEFUNCTIONS OF THE DEFECT LEVELS ASSOCIATED WITH THE TRI-CARBON INTERSTITIAL CLUSTER

To elucidate the degree of localization of the defect states, we investigated the wavefunctions of the tri-carbon interstitial cluster. The tri-carbon interstitial cluster is embedded in a 576-atom 4H-SiC supercell. The Heyd, Scuseria, and Ernzerhof (HSE06) functional [1] is employed to calculate the wavefunctions using a single Γ-point. In this approach, we mix a portion of the nonlocal Hartree–Fock exchange into the generalized gradient approximation of Perdew, Burke, and Ernzerhof (PBE) [2], with the default fraction ($\alpha = 0.25$) and an inverse screening length of 0.2 Å^{-1} to accurately describe the electronic structure of 4H-SiC. The fully relaxed geometries were obtained by minimizing the quantum mechanical forces between the ions to below 0.01 eV/Å, and the self-consistent calculations were converged to 10^{-5} eV. The electron wavefunctions were expanded in a plane-wave basis set with a cutoff energy of 420 eV.

The tri-carbon interstitial cluster consists of three carbon interstitials bridging three adjacent on-axis Si-C bonds at the hexagonal site. In the ground state, the defect exhibits C_{3v} symmetry and is the most stable among all possible configurations [3]. The wavefunctions of the defect levels (DLs) and the band edges [valence band maximum (VBM) and conduction band minimum (CBM)] are illustrated in Supplementary Figure 1. The wavefunctions of the DLs are localized around the defect.

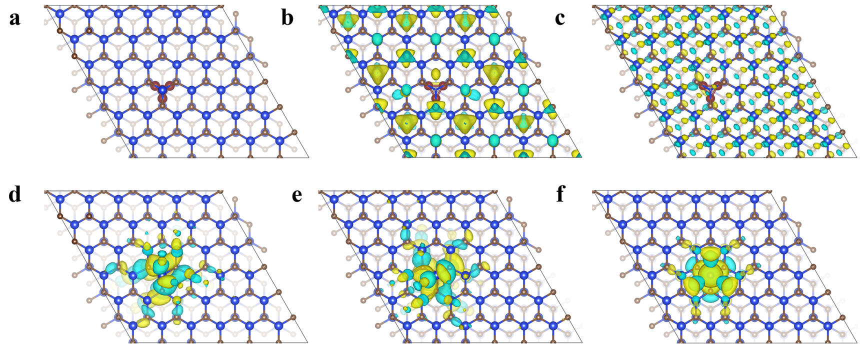

Supplementary Figure 1. Geometry and Kohn-Sham wavefunctions of the defect in the ground state with C_{3v} symmetry. **a** Geometry, **b** CBM wavefunction, **c** VBM wavefunction, **d**-**e** double degenerate e states and **f** non-degenerate a state. Isosurface value is set to 5×10^{-5} electron/bohr^3. The carbon interstitial atoms are represented by deep red spheres.

In calculating the excited state, we created a hole on the double-degenerate e state. The relevant parameters in the INCAR file are configured as follows:

FERWE = 1154*1.0 1*0.0 1*1.0 1*1.0 1*1.0 1*1.0 1200*0.0 # The syntax is BANDS*OCC spin-up,

FERDO = 1154*1.0 1*1.0 1*1.0 1*1.0 1*1.0 1*0.0 1200*0.0 # The syntax is BANDS*OCC spin-dw.

During optical excitation, an electron is promoted from the double-degenerate e state to the CBM, which gives rise to the so-called dynamical Jahn-Teller effect [4]. Accordingly, the symmetry is reduced from C_{3v} to C_{1h} (Supplementary Figure 2). In the excited state, the hole DL appears in the band gap at 0.07 eV above the VBM (see Supplementary Table I) in the spin-polarized ΔSCF DFT calculation. The wavefunctions of the hole DL and band edges are illustrated

in Supplementary Figure 3. The hole wavefunction is localized around the defect.

Similar to the method used for calculating the excited state, we also created a hole on the double-degenerate e state for the positively charged state. The relevant parameters in the INCAR file are configured as follows:

FERWE = 1154*1.0 1*0.0 1*1.0 1*1.0 1*1.0 1*0.0 1200*0.0 # The syntax is BANDS*OCC spin-up,

FERDO = 1154*1.0 1*1.0 1*1.0 1*1.0 1*1.0 1*0.0 1200*0.0 # The syntax is BANDS*OCC spin-dw.

In the positively charged state, the defect also exhibits C_{1h} symmetry, with the hole DL appearing in the band gap at 0.07 eV above the VBM (see Supplementary Table I), consistent with the excited state results. The wavefunctions of the hole DL and band edges are shown in Supplementary Figure 3.

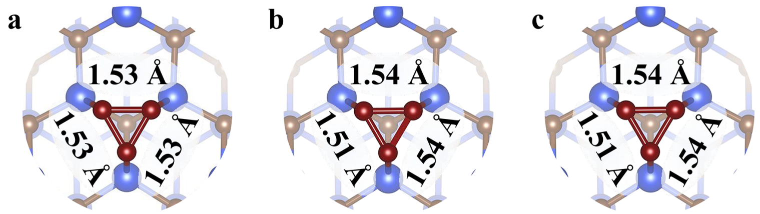

Supplementary Figure 2. The local geometries of the defect in **a** ground state, **b** excited state, and **c** positively charged state.

Supplementary Table I. The energies of the KS defect levels (DLs) in the ground state, excited state, and positively charged state.

		ground state	excited state	positively charged state
spin up	e state	VBM - 0.15 eV	VBM + 0.07 eV (**hole**)	VBM + 0.07 eV (**hole**)
	e state	VBM - 0.15 eV	VBM - 0.42 eV	VBM - 0.42 eV
	a state	VBM - 0.20 eV	VBM - 0.33 eV	VBM - 0.33 eV
			CBM - 0.06 eV (**electron**)	
spin dw	e state	VBM - 0.15 eV	VBM - 0.45 eV	VBM - 0.45 eV
	e state	VBM - 0.15 eV	VBM - 0.46 eV	VBM - 0.46 eV
	a state	VBM - 0.20 eV	VBM - 0.40 eV	VBM - 0.39 eV

SUPPLEMENTARY NOTE 2: THE EFFECT OF THE TRI-CARBON INTERSTITIAL CLUSTER ON VBM

The shape of the band edges predominantly influences the behavior of charge carriers and their interactions within the semiconductor. To assess the influence of DLs on the VBM, we conducted a comparative analysis of the band structure without and with the tri-carbon interstitial cluster in a 576-atom 4H-SiC supercell by HSE06 [1] at the neutral ground state. The band structures are calculated using the band unfolding method [5, 6]. In the calculation, we focused on two high symmetry points, Γ and M, and included 30 k-points along each high symmetry line.

The result, depicted in Supplementary Figure 4, demonstrates that despite the existence of DLs, the VBM remains

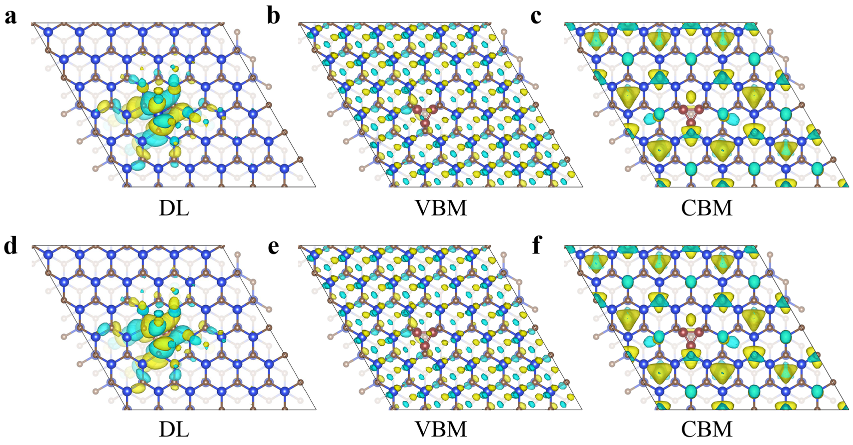

Supplementary Figure 3. Geometry and Kohn-Sham wavefunctions of the defect. **a-c** wavefunctions of excited state. **d-f** wavefunctions of positively charged state. Isosurface value is set to $5{\times}10^{-5}$ electron/bohr^3. The carbon interstitial atoms are represented by deep red spheres.

remarkably unaltered at the Γ-point (Supplementary Figure 4b). Our observations strongly indicate that the DLs have limited influence on the shape of the top bands in the energy region close to the VBM.

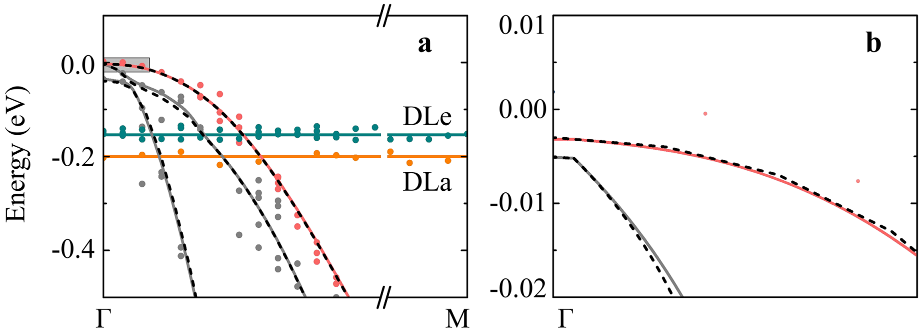

Supplementary Figure 4. Band structure of the defective 4H-SiC at the neutral ground state. **a** The unfolded band structure of 4H-SiC without (dashed lines) and with (solid lines) the tri-carbon interstitial cluster. **b** The partial enlarged view of the band structure at Γ-point. The DLe and DLa are obtained by fitting data to the green and yellow points, respectively. The standard deviations are 0.007 eV and 0.009 eV, respectively.

SUPPLEMENTARY NOTE 3: THE CHARGE TRANSITION LEVEL OF THE TRI-CARBON INTERSTITIAL CLUSTER

The formation energies E_f of defects are calculated to determine the charge stability with the following equation [7, 8],

$$E_f^q = E_d^q - E_{\text{host}} + \sum n_i(\Delta\mu_i + E_i) + q\left(E_{\text{VBM}} + E_{\text{Fermi}}\right) + E_{\text{corr}}\left(q\right), \tag{1}$$

where E_d^q is the total energy calculated with a supercell containing a defect in a charge state q. E_{host} is the total energy for the defect-free supercell, whereas n_i is the number of atoms removed from or added to the supercell. $\Delta\mu_i$ is the chemical potential of constituent i referenced to bulk silicon and diamond with energy E_i. In this paper, we only consider the carbon-rich condition and the values of $\Delta\mu_{\text{C}}$ and $\Delta\mu_{\text{Si}}$ are -11.33 eV and -6.14 eV. E_{VBM} and E_{Fermi} are the energy of the VBM and Fermi level referenced to the VBM of the host, respectively. The $E_{\text{corr}}(q)$ is the correction term for the charged system due to the existence of electrostatic interactions of the periodic images of the defect. We used the correction method of Freysoldt, Neugebauer, and Van de Walle (FNV) [9] for the total energy of the defective systems. We obtained 0.099 eV for this system.

The charge transition level $\epsilon_d^{q/q'}$ can be derived from Eq.(1) as follows

$$\epsilon_d^{q/q'} = (E_f^q - E_f^{q'})/(q' - q), \tag{2}$$

where E_f^q and $E_f^{q'}$ are the formation energy of the defect in charge state q and q', respectively.

For the tri-carbon interstitial cluster, the calculated formation energy is 12.02 eV. The charge-state transition level is located below the VBM (VBM$-$0.22 eV, as shown in Supplementary Figure 5), indicating that the positively charged state of this defect is not thermodynamically stable. As anticipated, the formation energy is relatively high, indicating that this defect is unlikely to form during equilibrium growth. However, experimental evidence suggests that such defects can arise in damage centers through non-equilibrium processes, such as carbon implantation, electron irradiation followed by annealing, or potentially through oxidation-induced injection of carbon interstitials [10, 11]. Therefore, the Si-rich or C-rich nature of the host is unlikely to significantly impact the defect formation process.

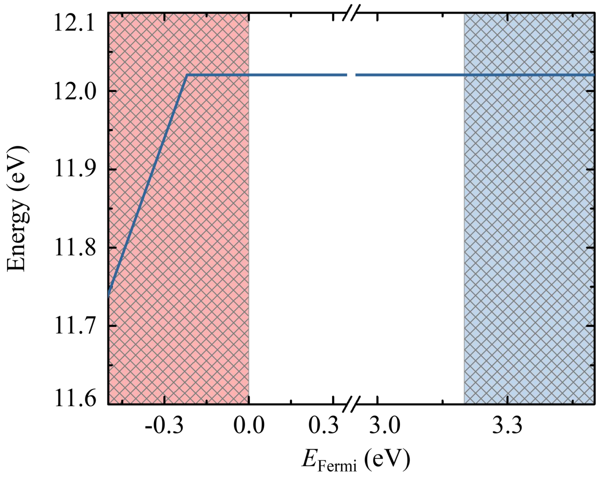

Supplementary Figure 5. Formation energy (E_f) of the tri-carbon interstitial cluster as a function of the Fermi level (E_{Fermi}).

SUPPLEMENTARY NOTE 4: OPTICAL SPECTRUM

GW approximation with Bethe-Salpeter equation (BSE) [12–14] is employed to calculate the exciton effect. We here use the supercell with 576-atom in order to reach close-to-converged calculation for the GW method which

is very computationally demanding in the VASP implementation as more than 9000 bands were included in the single-shot G_0W_0 calculation. The energy cutoff for the response function is set to be 150 eV. The Tamm-Dancoff approximation was used to solve BSE. The highest one hundred valence bands and one hundred lowest conduction bands are considered as the basis for the excited state in the BSE procedure. The calculations are based on the standard HSE06 functional which resulted in the optimized geometry and the electronic structure.

The band structure of perfect 4H-SiC is indirect. The VBM is located in the Γ-point where the crystal field splits the threefold band into a twofold degenerate band and a non-degenerate band. The CBM is located in the M-point which transforms as M_4 in the C_{2v} space group. Because of the three equivalent CBM valleys, this state will be threefold degenerate in the calculations. There is a second conduction band in the M-point lying above with about 140 meV that transforms as M_1 in the C_{2v} space group, and it is again threefold degenerate. The next local minimum in the conduction band can be found in the L-point which resides about 300 meV above the M_1 band minimum. The L-point is also threefold degenerate. We emphasize that all these critical k-points are projected to the Γ-point of the applied supercell.

Supplementary Figure 6 displays the nature of the exciton and the oscillator strength of the optical transitions in perfect 4H-SiC by BSE method. The distinct peak at 4.31 eV signifies the minimum threshold energy for the optical transition, mainly attributed to the electron excitation between the band edges located at the M-point within the Brillouin zone of 4H-SiC.

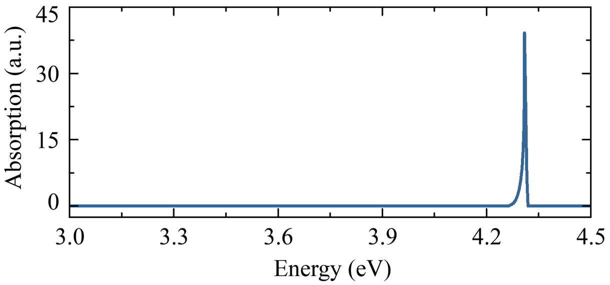

Supplementary Figure 6. The optical transition intensity of 4H-SiC calculated by the BSE method.

In the defective supercells DLs and resonant states (R) occur. Resonant states are such states where the defect states are mixed with the valence bands having similar energies, and the respective wavefunctions are quasilocalized around the core of the defect. The defective potential splits the threefold degenerate conduction bands to a twofold degenerate and a non-degenerate conduction band in the M-point and L-point. It is highly likely that the pseudo-donor character of the exciton will be developed at the M-point of CBM, thus the analysis of the low-energy BSE spectrum does have a direct relevance in the interpretation of the photoluminescence signal. Nevertheless, we also analyze here the observed peaks at higher energies for the sake of completeness.

The contribution of the quasiparticle states and the oscillator strength of the optical transitions within the tri-carbon interstitial cluster are shown in Supplementary Figure 7. In the energy range spanning 3.15 eV to 3.20 eV, one double degenerate peak (P_1 in Supplementary Figure 7b) and one non-degenerate peak (P_2 in Supplementary Figure 7b) emerge. Both peaks primarily result from the excitation from DLs to the CBM, collectively constituting approximately

95% and 99% of the total intensity. The excitonic wavefunctions of P_1 and P_2 are depicted in Supplementary Figure 8. The double degenerate peak denoted as P_1 in Supplementary Figure 7b is primarily caused by the excitation from the e state (DLe) to CBM, constituting approximately 93.71%. The excitation from the a states (DLa) to CBM contributes about 1.40%. This result is convincing to apply ΔSCF calculation for the excited state where the electron is promoted from the DLe to CBM. The origin of the P_2 peak is depicted in Supplementary Figure 9a. The primary contribution stems from excitation originating from the non-degenerate DLa to the CBM, accounting for approximately 98.84% of the total intensity whereas the excitation from the DLe to the CBM negligibly contributes (0.05%). We note that both peaks suggest the pseudo-donor nature of the first bright excited state of the defect where the hole part of the exciton is localized to DLs whereas the electron is located at the M-point of the BZ.

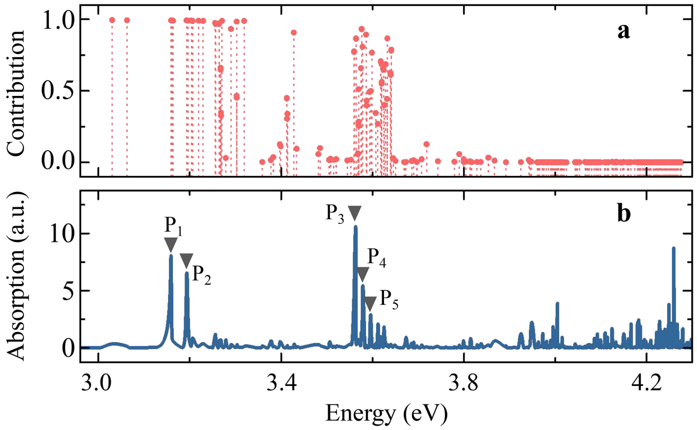

Supplementary Figure 7. Analysis and plot of the optical spectrum. **a** The total contributions of the excitation from DLs and resonant states (R) to CBM and the conduction bands in the L-point. **b** The optical transition intensity of the tri-carbon interstitial cluster.

In contrast to the previously discussed peaks, the peaks P_3 and P_4 in Supplementary Figure 7b are mainly caused by the excitation from DLs to the L-point in the conduction band. The origin of the P_3 peak is presented in Supplementary Figure 9b, with contributions of approximately 12.32% from excitation to the CBM and 86.93% from excitation to the L-point in the conduction band. Similarly, the P_4 peak is also predominantly resulted from the excitation to the L-point in the conduction bands, constituting approximately 82.03% as illustrated in Supplementary Figure 9c. For P_5 peak, the contribution from DLs and resonance states has been reduced to 50.44% (see Supplementary Figure 9d). For the peaks located at the energies exceeding 3.65 eV, the contribution of the excitation from DLs and resonance states to CBM diminishes to less than 10%.

SUPPLEMENTARY NOTE 5: THE CALCULATION OF ZPL

The zero-phonon-line (ZPL) energy (E_{ZPL}), depending on the electronic structure and ionic relaxation induced by excitation, is a criterion for potentially identifying an experimentally isolated emitter. Here, the E_{ZPL} is calculated

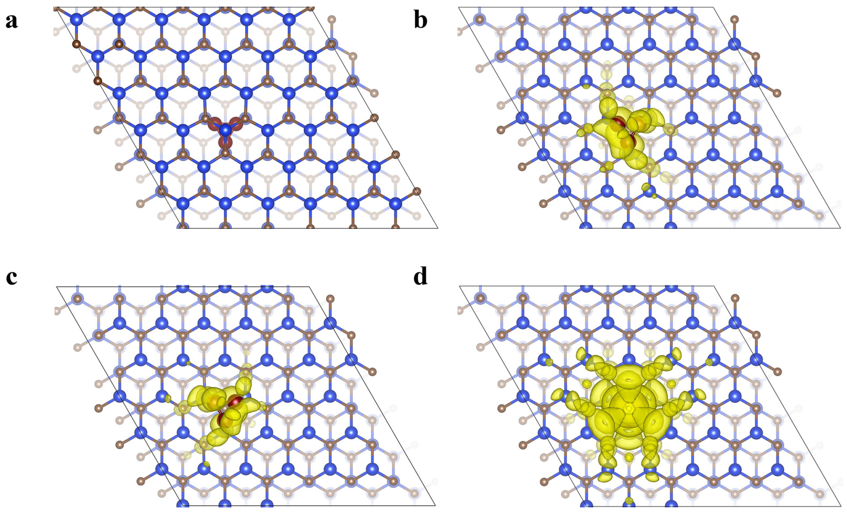

Supplementary Figure 8. Geometry and the wavefunctions of the exciton of the defect. **a** Geometry and the hole part of excitonic wavefunctions **b**-**c** for the double degenerate P_1 states and **d** non-degenerate P_2 state in Fig. 3 in the main text. Isosurface value is set to 5×10^{-4} electron/bohr^3.

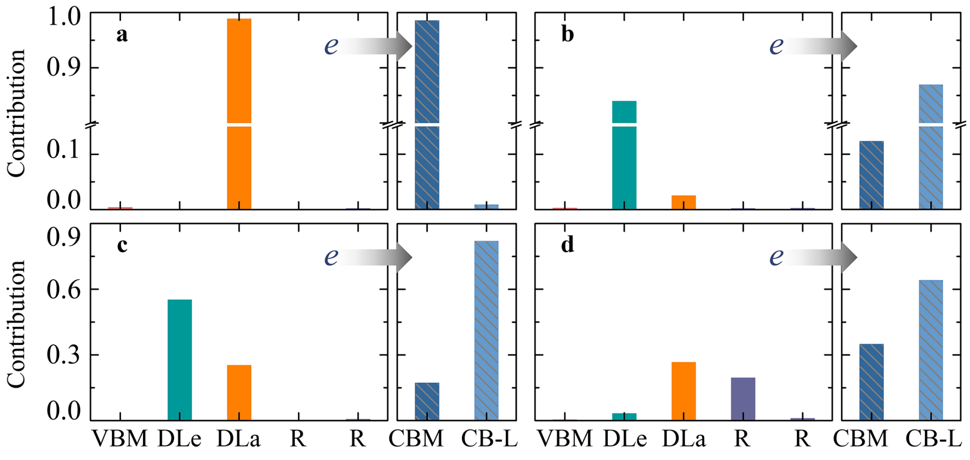

Supplementary Figure 9. Analysis of high-energy exciton states. The contributions of excitation originating from VBM, DLs, and resonances state (R) to CBM and conduction band in the L-point (CB-L) for the peaks **a** P_2, **b** P_3, **c** P_4 and **d** P_5 in Supplementary Figure 7.

with the following equation

$$E_{\text{ZPL}} = E_{\text{vertical}} - E_{\text{relaxation}}, \tag{3}$$

where E_{vertical} is the vertical excitation energy calculated within BSE [12–14]. For the tri-carbon interstitial cluster in the 576-atom 4H-SiC supercell, E_{vertical} is 3.16 eV. $E_{\text{relaxation}}$ is ionic relaxation induced by excitation in the ΔSCF calculation [15–17]. For the tri-carbon interstitial cluster, $E_{\text{relaxation}}$ is 0.13 eV.

One limitation of the ΔSCF approach is that it does not provide an exact representation of the true excited states, particularly in cases where the singlet excited state is not accurately captured. Notably, for the tri-carbon interstitial

cluster, the triplet state may provide a better approximation of the many-body singlet manifold, with its geometry offering a more reliable description of the excited state. In our calculations, the total energy of the triplet state is lower than that of the singlet excited state by 0.010 eV without relaxation and by 0.014 eV after relaxation. Given the small magnitude of this energy difference, its impact can be considered negligible.

In addition, we applied FNV corrections [9] considering the excited state has a pseudo-donor nature, i.e., the system can be considered as a positively charged core and a loosely bound electron orbiting in the effective mass state (∼0.1 eV). The background for the need of the size correction following the charge correction of a charged supercell is explained in section 3.3.4 of Ref. 18 and references therein which consider the bound exciton defects (e.g., W-center in silicon [19]). This size correction has been successfully applied to neutral silicon-vacancy in diamond [20] and the carbon-oxygen split interstitial cluster in silicon [21]. The final calculated E_{ZPL} is at 3.13 eV which falls below the calculated indirect band gap of 4H-SiC.

SUPPLEMENTARY NOTE 6: LVMS OF THE TRI-CARBON INTERSTITIAL CLUSTER

Previously, the tri-carbon interstitial cluster has been suggested as the possible source of the $\mathrm{D_{II}}$ photoluminescence (PL) spectra, based on calculated local vibration modes (LVMs) as discussed in Ref. 22. For the vibrational modes, we calculated the corresponding dynamical matrix containing the second order derivatives of the total energy by means of the PBE [2] functional where all the atoms in the supercell were enabled to vibrate. In this case, we apply strict threshold parameters for the convergence of the electronic structure (10^{-6} eV) and atomic forces (10^{-3} eV/Å) in the geometry optimization procedure. These vibration modes are applied together with the HSE06 ground state and excited state geometries to simulate the effective LVMs and PL spectrum of the given defects within Huang-Rhys theory. This strategy worked well for deep defects in diamond (e.g., Refs. 23 and 24).

We found that the calculated ZPL of the tri-carbon interstitial cluster is 3.13 eV. This calculation aligns well with the experimental measurements reported in Refs. 11 and 25. The partial Huang-Rhys factors (HR-factors) and LVMs of tri-carbon interstitial cluster are presented in Supplementary Figure 10 and Supplementary Table II. The vibration modes with the frequency larger than that of 4H-SiC bulk phonon spectrum (115.0 meV) in Supplementary Figure 10 correspond to effective LVMs during the excitation, indicating coupling between the electronic excited state and specific vibrational modes. The vibrations of those LVMs are shown in Supplementary Figure 11. The double degenerate highest LVMs at 162 meV are stretching modes from two of the three approximately vertical C-C bonds (see Supplementary Figures 11a,b). The third LVM is a stretching mode of the three C-C bonds, which preserves the symmetry of the defect (see Supplementary Figure 11c). The fourth LVM is a breathing mode of the triangle formed by the three carbon interstitials, shown in Supplementary Figure 11d. The respective fifth and sixth LVMs result from the axial vibration of the carbon atoms right below and above the center of the tri-carbon interstitial cluster (see Supplementary Figures 11e,f). The vibration modes with the frequency close to that of SiC bulk phonon mode distort the symmetry of the geometry (see Supplementary Figures 11g,h).

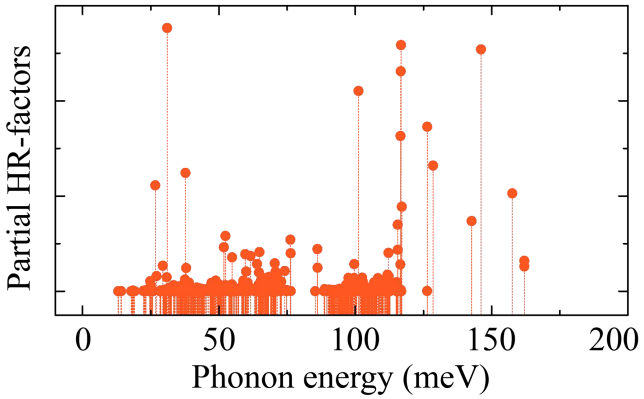

Supplementary Figure 10. The partial Huang-Rhys factors (HR-factors) of tri-carbon interstitial cluster.

Supplementary Table II. The comparison of energies of ZPLs (E_{ZPL}) together with their effective LVMs during the excitation between the previously reported $\mathrm{D_{II}}$ center [11, 25] and our calculated results of the tri-carbon interstitial cluster in 4H-SiC.

	E_{ZPL} (eV)	LVM (meV)									
$\mathrm{D_{II}}$ center [11]	3.20	164.1	160.0	156.8	153.9	148.7	145.9	141.9	137.1	126.9	123.8
$\mathrm{D_{II}}$ center [25]	3.21	164.4		152.4			146.1		129.8	127.1	
tri-carbon interstitial cluster	3.13	162.0 (2×)		157.6			146.1	142.7	128.6	126.4 (2×)	

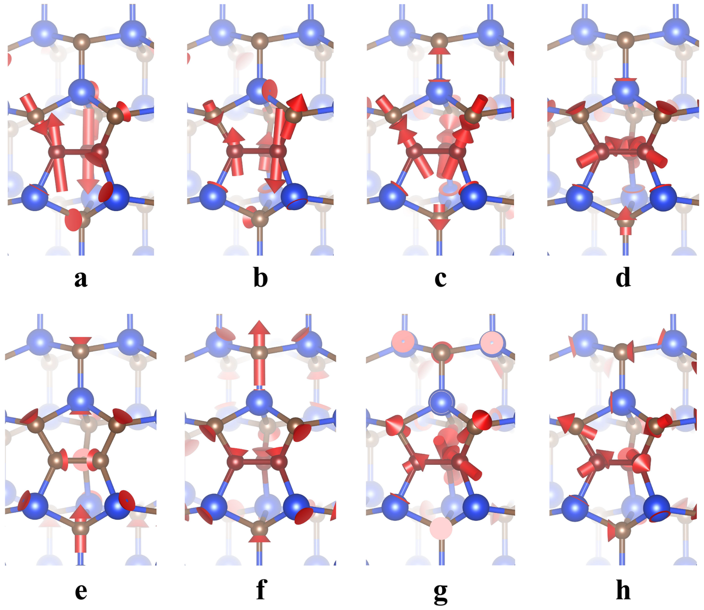

Supplementary Figure 11. The vibrations of LVMs with frequencies higher than that of 4H-SiC bulk phonon spectrum. **a** and **b** depict the degenerate 162.0-meV modes, **c**, **d**, **e** and **f** depict 157.6-meV, 146.1-meV, 142.7-meV, and 128.6-meV modes, respectively, whereas **g** and **h** depict the 126.4-meV degenerate modes.

[1] J. Heyd, G. E. Scuseria, and M. Ernzerhof, The Journal of Chemical Physics **118**, 8207 (2003).

[2] J. P. Perdew, K. Burke, and M. Ernzerhof, Physical Review Letters **77**, 3865 (1996).

[3] P. Li, P. Udvarhelyi, S. Li, B. Huang, and A. Gali, Physical Review B **108**, 085201 (2023).

[4] M. Sturge, in *Solid state physics*, Vol. 20 (Elsevier, 1968) pp. 91–211.

[5] V. Popescu and A. Zunger, Physical review letters **104**, 236403 (2010).

[6] V. Popescu and A. Zunger, Physical Review B **85**, 085201 (2012).

[7] S. B. Zhang and J. E. Northrup, Physical Review Letters **67**, 2339 (1991).

[8] C. Freysoldt, B. Grabowski, T. Hickel, J. Neugebauer, G. Kresse, A. Janotti, and C. G. Van de Walle, Reviews of Modern Physics **86**, 253 (2014).

[9] C. Freysoldt, J. Neugebauer, and C. G. Van de Walle, Physical Review Letters **102**, 016402 (2009).

[10] L. Patrick and W. Choyke, Journal of Physics and Chemistry of Solids **34**, 565 (1973).

[11] W. Sullivan and J. W. Steeds, in *Materials Science Forum*, Vol. 556 (Trans Tech Publ, 2007) pp. 319–322.

[12] K. Krause and W. Klopper, "Implementation of the bethe-salpeter equation in the turbomole program," (2017).

[13] M. Shishkin and G. Kresse, Physical Review B **74**, 035101 (2006).

[14] M. Shishkin, M. Marsman, and G. Kresse, Physical Review Letters **99**, 246403 (2007).

[15] A. Gali, E. Janzén, P. Deák, G. Kresse, and E. Kaxiras, Physical Review Letters **103**, 186404 (2009).

[16] R. O. Jones and O. Gunnarsson, Reviews of Modern Physics **61**, 689 (1989).

[17] J. Gavnholt, T. Olsen, M. Engelund, and J. Schiøtz, Physical Review B—Condensed Matter and Materials Physics **78**, 075441 (2008).

[18] Á. Gali, Nanophotonics **12**, 359 (2023).

[19] Y. Baron, A. Durand, P. Udvarhelyi, T. Herzig, M. Khoury, S. Pezzagna, J. Meijer, I. Robert-Philip, M. Abbarchi, J.-M. Hartmann, *et al.*, ACS photonics **9**, 2337 (2022).

[20] Z.-H. Zhang, P. Stevenson, G. Thiering, B. C. Rose, D. Huang, A. M. Edmonds, M. L. Markham, S. A. Lyon, A. Gali, and N. P. de Leon, Physical Review Letters **125**, 237402 (2020).

[21] P. Udvarhelyi, A. Pershin, P. Deák, and A. Gali, npj Computational Materials **8**, 262 (2022).

[22] C. Jiang, D. Morgan, and I. Szlufarska, Physical Review B **86**, 144118 (2012).

[23] G. Thiering and A. Gali, Physical Review B **96**, 081115 (2017).

[24] G. Thiering and A. Gali, Physical Review X **8**, 021063 (2018).

[25] S. Sridhara, D. Nizhner, R. P. Devaty, W. J. Choyke, T. Dalibor, G. Pensl, and T. Kimoto, in *Materials Science Forum*, Vol. 264 (Trans Tech Publ, 1998) pp. 493–496.
